# Case Report: Hypertensive encephalopathy presents with complex types of sleep breathing events

**DOI:** 10.3389/fcvm.2025.1742605

**Published:** 2026-01-12

**Authors:** Yunchao Huang, Huiling Luo, Chunmei Pan, Qifen Jiang, Bin Ma

**Affiliations:** 1 Department of Respiratory and Critical Care Medicine, The Affiliated Hospital of Yunnan University, Kunming, China; 2 School of Basic Medical Sciences, Lanzhou University, Lanzhou, China; 3 Department of Respiratory Medicine II, The Second People's Hospital of Lanzhou City, Lanzhou, China; 4 Research Center for Medical Device Regulatory Science, Lanzhou University, Lanzhou, China; 5 Industry Center for Evidence-Based Research and Evaluation Standards in Medical Devices, Lanzhou, Gansu, China

**Keywords:** hypertension, hypertensive encephalopathy, non-invasive ventilation, polysomnography, sleep apnea

## Abstract

**Background:**

Obstructive sleep apnea (OSA) is a common sleep disorder and one of the common causes of secondary hypertension. Patients with this condition often have characteristics such as reverse spoon-shaped blood pressure patterns and poor response to multiple drug treatments. Patients often attribute morning dizziness and fatigue to poor sleep quality, while neglecting the diagnosis and control of hypertension. Hypertensive encephalopathy (HE) is a hypertensive emergency, which may present with symptoms such as headache, vomiting, and epileptic-like seizures due to increased intracranial pressure. The impact of hypertension on sleep has not been reported in the literature.

**Case summary:**

A 43-year-old male patient complained of having experienced elevated blood pressure for two years, and his blood pressure was poorly controlled without any monitoring. The patient had no family history of hypertension, and found that the blood pressure was not controlled after increasing blood pressure, and then came to the hospital for treatment due to the sudden increase of blood pressure, accompanied by dizziness and headache. During the hospitalization, the patients used various drugs with poor antihypertensive effect, and after examining the hypertension-related causes, it pointed to sleep dyspnea-related hypertension. During this period, the patient developed HE, sleep breathing disorder changed from obstructive to central, and tidal breathing appeared at the same time. After various non-invasive ventilator modes and pressure titration, the patient was treated and discharged from hospital.

**Conclusion:**

Patients with HE may experience complex types of sleep breathing disorders, which further lead to intermittent or persistent hypoxemia, making it difficult to control blood pressure and potentially damaging various organs throughout the body. During the period of providing respiratory support for such patients, it is necessary to pay attention to the supply of oxygen and the setting of ventilation modes for the current sleep breathing disorders. In cases where necessary, a multidisciplinary collaborative diagnosis involving sleep experts, respiratory experts, and neurology experts should be conducted.

## Graphical Abstract

**Figure:**
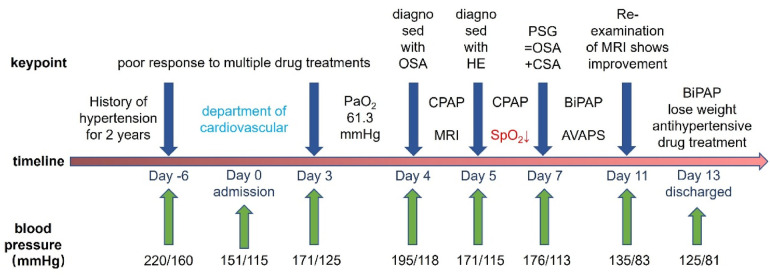
Disease diagnosis and treatment process and key points

## 1 Introduction

Obstructive sleep apnea (OSA) is a prevalent etiology of secondary hypertension, often accompanied by symptoms such as dizziness, headache, fatigue, and other manifestations. Blood pressure elevation primarily occurs during the night and is particularly pronounced in the morning hours. In clinical practice, these patients tend to overlook blood pressure monitoring due to their younger age group. Consequently, most seek medical attention only when they experience severe clinical symptoms or abnormal blood pressure readings are detected during physical examination. Some individuals may present with hypertensive encephalopathy (HE) upon seeking emergency treatment. HE refers to a series of transient cerebral circulation dysfunctions caused by cerebral edema, increased intracranial pressure, and even herniation resulting from sudden elevation of blood pressure beyond the threshold for automatic regulation of cerebral blood flow. It is a critical condition that poses life-threatening risks for hypertensive patients and represents one of the common emergencies encountered in internal medicine departments.

This case report describes the comprehensive management approach employed for a patient presenting with HE complicated by respiratory failure associated with OSA. Following the occurrence of HE, sleep breathing monitoring was conducted revealing significant central sleep apnea (CSA) events. Subsequently, non-invasive mechanical ventilation was applied along with parameter adjustments leading to successful discharge from hospital care. During treatment for HE cases like this one, close attention should be paid to respiratory conditions as types of sleep-breathing events may vary necessitating real-time adjustment in respiratory support.

## 2 Case presentation

A 43-year-old male administrative worker was admitted to the Department of Cardiovascular Medicine in October 2023 with a chief complaint of “elevated blood pressure discovered 2 years prior.” The patient had a 2-year history of hypertension without regular blood pressure monitoring and had discontinued antihypertensive medications one year prior to admission; details of his medication regimen were unclear. One week before admission, he experienced sudden onset of dizziness, headache, vertigo, and chest tightness, with blood pressure measured at 220/160 mmHg. Despite treatment with multiple antihypertensive agents at an outside hospital, blood pressure control remained poor, and he was transferred to our cardiovascular department via emergency services with a diagnosis of “hypertensive crisis”. The patient denies any previous medical history and family history of hereditary diseases, smoked for 10 years, 5–10 cigarettes per day, with occasional light alcohol consumption. Physical examination revealed: body temperature 36.2°C, respiratory rate 20 breaths/min, pulse 97 beats/min, blood pressure 151/115 mmHg (1mmHg = 0.133KPa), body weight 97 kg, and BMI 31.67 kg/m^2^. General condition was fair with clear consciousness and mild cyanosis of the lips. No rales were auscultated in either lung field. Cardiac dullness was enlarged to the left, heart rate was 97 beats/min with regular rhythm, and no murmurs, extra heart sounds, or pericardial friction rubs were detected. The abdomen was soft without tenderness, and there was no lower extremity edema. Following admission, the patient received oral nifedipine controlled-release tablets 30 mg twice daily, metoprolol succinate extended-release tablets 23.75 mg once daily, terazosin hydrochloride capsules 2 mg at bedtime, plus continuous intravenous infusion of urapidil for blood pressure control. Despite this regimen, blood pressure continued to fluctuate between 145 and 195/95–125 mmHg. Relevant abnormal laboratory findings included: 8 AM cortisol + adrenocorticotropic hormone (ACTH): aldosterone (supine position) 17.09 ng/dL(1–16), thyroid function test: serum thyroid-stimulating hormone 4.58 μIU/mL (1.24–3.62), sex hormone panel: serum prolactin 25.59 ng/mL (2.64–13.13). Serum electrolytes were normal, other blood tests were within normal limits (Table 1), Urinalysis, stool examination, abdominal ultrasonography and cranial computed tomography (CT) were unremarkable. After reviewing these results, renal, endocrine, neurogenic, and major vascular causes of secondary hypertension were excluded. Despite multiple adjustments to antihypertensive medications following admission, blood pressure control remained suboptimal. Arterial blood gas analysis was subsequently performed, revealing: partial pressure of oxygen (PaO_2_) 61.3 mmHg and partial pressure of carbon dioxide (PaCO_2_) 31.6 mmHg, Chest CT and pulmonary artery CTA did not show any abnormalities. Given the patient's obesity and history of nocturnal snoring, the Epworth score was 12, and the STOP-BANG score was 6, a sleep breathing monitoring screening test was performed, which showed: apnea-hypopnea index (AHI) 47.6 events/h, obstructive sleep apnea (OSA) 45.6 events/h, oxygen desaturation index 67.2 events/h, and minimum oxygen saturation 44%. These findings indicated severe sleep apnea-hypopnea syndrome, predominantly obstructive type, with severe hypoxemia, making it impossible to perform continuous positive airway pressure (CPAP) pressure titration under PSG monitoring. It is considered appropriate to first use automatic CPAP (AUTO-CPAP) to identify an appropriate pressure range and median pressure, and then gradually proceed with slow pressure titration. Additionally, under AUTO-CPAP treatment, the improvement in daytime oxygenation was insufficient, so supplemental oxygen therapy was added. During treatment, the patient continued to experience recurrent dizziness and headache with poor blood pressure control. A subsequent brain MRI revealed bilateral symmetric patchy abnormal signals in the periventricular white matter, centrum semiovale, and cerebellar hemispheres, suggestive of cerebral edema. Two abnormal signal nodules in the right parietal lobe and punctate abnormal signals in the pons and right cerebellar hemisphere were consistent with HE. Treatment with multiple antihypertensive agents combined with measures to reduce cerebral edema was continued.

**Table 1:** The blood test results for hypertension-related hormone levels.

Test Item	Result	Reference range	Unit
Cortisol (8:00)	185.19	42.6–248.5	ng/mL
Cortisol (24:00)	45.31	42.6–248.5	ng/mL
Adrenocorticotropic hormone (8:00)	39.49	7.2–63.4	pg/mL
Adrenocorticotropic hormone (24:00)	7.6	7.2–63.4	pg/mL
Aldosterone (recumbent)	17.09*	1–16	ng/dL
Renin (recumbent)	21.14	2.4–32.8	ng/L
Angiotensin II (recumbent)	103.08	25–129	pg/mL
Aldosterone/Renin ratio	0.81	<5.7	–
Serum Prolactin (PRL)	25.59*	2.64–13.13	ng/mL
Testosterone (T)	347.18	175–781	ng/dL
Serum Growth Hormone (HGH)	0.34	0.030–2.47	ng/ml
Progesterone (P)	0.58	0.14–2.06	ng/ml
Follicle-Stimulating Hormone (FSH)	5.12	1.27–19.26	mIU/mL
Luteinizing Hormone (LH)	4.58^a^	1.24–3.62	mIU/mL
Estradiol (E2)	29.07	15.16–38.95	pg/m
Total Triiodothyronine (TT3)	1.21	0.8–2.0	nmol/L
Total Thyroxine (TT4)	103.2	66–181	nmol/L
Free Triiodothyronine (FT3)	3.5	2.1–5.4	pmol/L
Free Thyroxine (FT4)	12.73	9.0–23.0	pmol/L
Thyroid-Stimulating Hormone (TSH)	3.36	0.35–5.10	mIU/L
Metanephrine(MN)	34	<90	pg/mL

a Indicating abnormal results.

**Figure 1:**
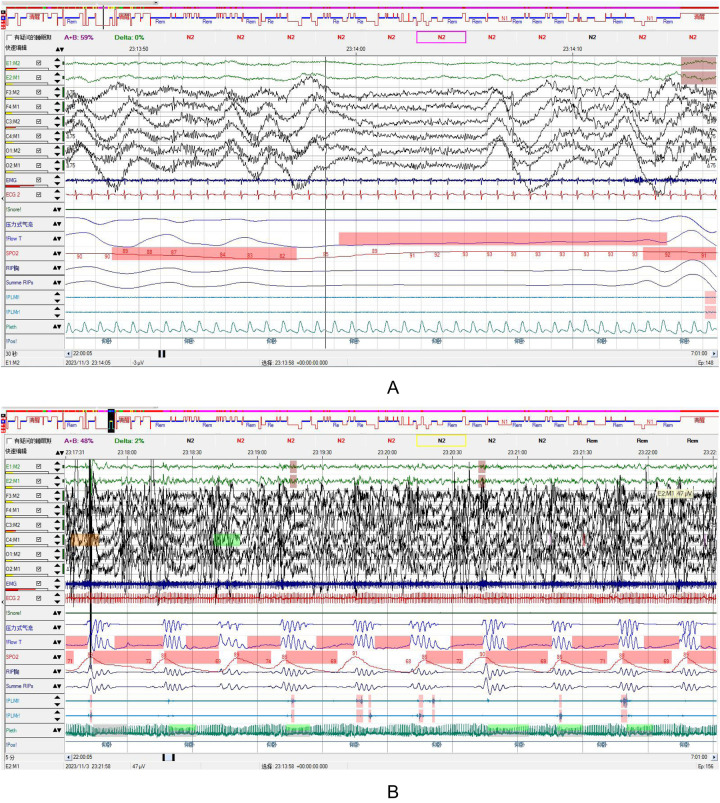
**(A,B)** The patient underwent sleep monitoring using the SOMNOscreen plus PSG+ system. The results indicated disrupted sleep architecture, with an increased duration of REM sleep and an absence of N3 stage sleep. Airflow monitoring revealed the presence of sleep apnea episodes accompanied by oxygen desaturation during respiratory events, with no significant changes in heart rate observed. Based on the thoracoabdominal movement patterns, a diagnosis of central sleep apnea-hypopnea syndrome was established. **(A)** Presented in 30 s epochs, clearly demonstrates the patient's sleep stages. **(B)** Displayed in 5 min epochs, provides a clear overview of the sleep respiratory events and corresponding oxygen desaturation.

**Figure 2:**
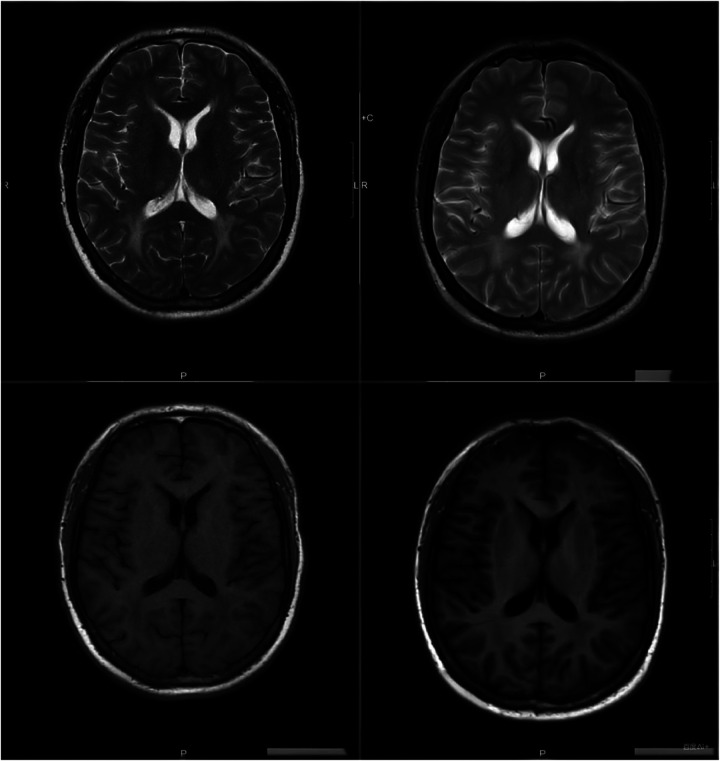
Comparison of head MRI findings before and after inpatient treatment: 1. The bilateral lateral ventricle, half oval center, corona radial, and bilateral frontoparietal lobe. 2. Puntate abnormal signals in the bridge and right cerebellar hemisphere, same with the former, considering the possibility of old bleeding lesions; 3. Small patchy abnormal signal lesions in bilateral basal ganglia and left lateral ventricle, same with the former, considering old lacunar infarction lesions.

One week after admission, the patient reported mild improvement in headache and dizziness but continued to experience palpitations and chest tightness with activity. Oxygen saturation ranged from 75%–85% without supplemental oxygen, and the hypoxemia remained unexplained. Additionally, irregular breathing rhythm was observed despite AUOT-CPAP therapy and supplemental oxygen, and blood pressure continued to fluctuate significantly at the upper limit of normal. After pulmonary embolism was excluded, the possibility of complex sleep-disordered breathing was considered. Once the patient's condition stabilized, a comprehensive polysomnography (PSG) study was performed, revealing: AHI 91.7 events/h, OSA 7.8 events/h, central sleep apnea (CSA) 23 events/hour, mixed sleep apnea (MSA) 2 events/h, hypopnea 58.2 events/h, oxygen desaturation index 95.7 events/h, minimum oxygen saturation 60%, mean oxygen saturation 82%, with Cheyne-Stokes respiration observed (see Figures 1, 2).

The patient's sleep-disordered breathing had evolved from purely obstructive events to a combination of obstructive and central sleep apnea-hypopnea syndrome, likely related to HE. Following aggressive treatment with measures to reduce intracranial pressure, intravenous aminophylline. Given the patient's inadequate improvement in oxygenation after CPAP treatment, there may be an underlying central respiratory issue that remains unaddressed. Adjusting the ventilator mode to bilevel positive airway pressure with Spontaneous/Timed (BiPAP ST) and incorporating average volume-assured pressure support (AVAPS) along with supplemental oxygen therapy could be considered (Table 2), the patient experienced significant improvement in dizziness and headache, blood pressure stabilization, and marked increase in oxygen saturation. Follow-up brain MRI with diffusion-weighted imaging (DWI) showed bilateral symmetric patchy abnormal signal foci in the periventricular white matter, centrum semiovale, corona radiata, and frontoparietal white matter, with reduced extent compared to prior imaging. Punctate abnormal signals in the pons and right cerebellar hemisphere were unchanged, suggestive of old hemorrhagic foci. Small patchy abnormal signal foci in the bilateral basal ganglia and left periventricular region were unchanged, consistent with old lacunar infarcts (Figures 2). Given the improvement in intracranial pathology, the patient was discharged. Before discharge, in order to reduce the patient's out-of-pocket cost for a home ventilator, the mode was adjusted to BiPAP ST mode. After using, the patient's vital signs remained stable, and sleep-disordered breathing events were at a relatively low level. Since the intracranial condition and its potential long-term impact on CSA have not been determined, the CPAP mode was not cautiously switched. The patient was advised to follow up regularly after discharge. Three telephone follow-up contacts revealed that the patient was using antihypertensive medications regularly and non-invasive ventilation during sleep. Blood pressure remained stable, nocturnal sleep quality was satisfactory, and nocturnal snoring with daytime fatigue and somnolence had significantly improved.

**Table 2:** AHI and blood oxygen changes after non-invasive ventilator treatment.

Time	Ventilator parameters (5 L/min oxygen intake)				Sleep apnea monitoring			
	Model	Pressure(cmH_2_0)		Tidal volume(ml)	AHI (/hr)	The lowest SpO_2_	Average oxygen full	Oxygen-minus index(/hr)
Hospital Day 4	Auto-CPAP	4–16		/	32.1	0.6	0.801	86.5
Hospital Day 7	BiPAP ST	IPAP 8	EPAP4	/	19.9	0.67	0.827	92.6
Hospital Day 8	AVAPS	IPAP6-12	EPAP5	450	8.3	0.66	0.835	91.6
Hospital Day 11	BiPAP ST	IPAP 10	EPAP4	/	11.6	0.81	0.966	8.3

AUTO CPAP, automatic continuous positive airway pressure; BiPAP ST, bilevel positive airway pressure spontaneous/timed; AVAPS, average volume-assured pressure support; IPAP, inspiratory positive airway pressure; EPAP, expiratory positive airway pressure.

## 3 Discussion

OSA is a common sleep-disordered breathing condition characterized by recurrent upper airway collapse during sleep, leading to chronic intermittent hypoxemia that results in daytime somnolence, cardiopulmonary and cerebrovascular complications, metabolic syndrome, and multi-organ damage (1). Multiple studies have demonstrated a close association between OSA and hypertension. Statistics indicate that 30%–40% of hypertensive patients have OSA, while 35%–80% of OSA patients have hypertension (2). In obese populations, the correlation between OSAS and hypertension is even more pronounced, and OSA of different phenotypes and severities are all associated with hypertension (3). Even mild OSAS demonstrates a dose-response relationship with systemic arterial hypertension risk (4). OSA-related hypertension is characterized primarily by onset in young adults, elevated blood pressure at night and upon morning awakening, disrupted blood pressure circadian rhythm, poor response to pharmacotherapy alone, and periodic blood pressure elevation accompanying apneic episodes. The chronic intermittent hypoxia induced by OSA leads to hypertension through pathophysiological changes including increased sympathetic nervous system excitability, endothelial damage, activation of the renin-angiotensin system (RAS), elevated serum catecholamine levels, increased endothelin release, and endothelial cell dysfunction (5). OSA occupies an important position among causes of secondary hypertension. Reviewing this patient's clinical course, he developed hypertension at a young age without a family history of hypertension, had irregular antihypertensive treatment, and poor blood pressure control. During hospitalization, a detailed differential diagnosis for secondary hypertension revealed no hormonal abnormalities. Multiple antihypertensive medications during hospitalization proved ineffective, and improvement in hypertension was only achieved after initiation of non-invasive ventilation therapy, which is consistent with characteristics of OSA-related hypertension. CPAP is the first-line treatment for OSA and can significantly reduce inflammatory mediators and oxidative stress in the blood of patients with OSA and hypertension. Furthermore, the therapeutic effect on hypertension in OSA patients shows a significant positive correlation with suppression of oxidative stress and inflammation (6). When OSA syndrome and hypertension coexist, comprehensive treatment should be adopted, with standard pharmacological therapy and non-invasive ventilation proceeding simultaneously (7). In clinical practice, cases of OSA patients achieving normal blood pressure after non-invasive ventilation therapy are occasionally encountered, which also demonstrates the therapeutic benefits of continuous positive airway pressure for patients with OSA-related hypertension.

Another distinctive feature of this patient was the development of HE in the context of elevated blood pressure maintained at the upper limit of normal. Previously, Edvardsson B reported a case of HE in the context of OSA, but sleep breathing monitoring was not performed during the period of HE (8). Untreated malignant hypertension typically leads to widespread vascular damage, potentially affecting many organs including the brain and kidneys, resulting in serious complications such as acute renal failure, microangiopathic hemolytic anemia, disseminated intravascular coagulation, and HE (9). HE accounts for 2% of acute hypertensive complications (10). During the development of HE, severely elevated blood pressure causes dysregulation of cerebral autoregulation, leading to increased intracranial pressure, which subsequently results in arteriolar vasodilation, cerebral edema, and petechial hemorrhage (9). Additionally, changes in intracranial carbon dioxide and hypoxia can directly dilate cerebral vessels, leading to severe cerebral edema, increased intracranial pressure, and increased incidence of cardiovascular and cerebrovascular diseases (11). Furthermore, the cerebrovascular regulatory system is impaired in many OSA patients under various chemical stimuli (12). This patient developed neurological symptoms within one week, and brain MRI revealed abnormal signals suggestive of cerebral edema and microhemorrhagic foci, confirming the diagnosis of HE. Sleep breathing monitoring also detected Cheyne-Stokes respiration and a significant proportion of central sleep apnea (CSA), both possibly related to elevated intracranial pressure and functional brainstem injury (13, 14). Following treatment with antihypertensive agents, measures to reduce intracranial pressure, and non-invasive ventilation, the patient's neurological symptoms improved. Repeat sleep breathing monitoring showed resolution of Cheyne-Stokes respiration and reduction in central sleep apnea, suggesting that HE may present with a series of respiratory changes (15). Obese OSA patients are more likely to experience a reduction in tidal volume, particularly when in a supine position. If respiratory rhythm disturbances are also present, the patient's minute ventilation may decrease sharply, posing a life-threatening risk. In such cases, when CPAP treatment is ineffective, it is important to initiate BiPAP-ST as early as possible to provide bilevel positive pressure support and control respiratory rate. If necessary, upgrading to the AVAPS mode should be considered to ensure adequate tidal volume, thereby reducing the risk of invasive mechanical ventilation. This indicates that close attention to respiratory status is necessary during treatment of HE to prevent respiratory failure that could endanger the patient's life.

In summary, young hypertensive patients without a family history should be promptly screened for concurrent OSA, while blood pressure should be monitored in OSA patients. Clinicians should remain vigilant for the development of HE in patients with uncontrolled hypertension. Patients who develop HE should be carefully monitored for possible sleep-disordered breathing events, with timely respiratory support provided and appropriate timing of sleep breathing monitoring determined. When initial CPAP therapy proves ineffective, attention should be paid to possible complex sleep-disordered breathing events for early detection and timely intervention. Early diagnosis of sleep-disordered breathing requires multidisciplinary collaborative care to achieve early recognition, early diagnosis, and early targeted treatment.

## 4 Patient perspective

The patient experienced a challenging but meaningful transformative process, evolving from initial questioning of the hypertensive etiology and diagnosis during early treatment, through critical illness with inadequate response to multiple medications, to clinical improvement after gradually accepting non-invasive ventilation therapy. The patient described this journey as one involving cognitive reappraisal and enhanced self-management awareness.

## Data Availability

The datasets presented in this study can be found in online repositories. The names of the repository/repositories and accession number(s) can be found in the article/Supplementary Material.
